# The CONSENSUS Study: Preliminary results of a mixed methods study to develop a core outcome set for late phase clinical trials in Oropharyngeal Cancer

**DOI:** 10.1186/1745-6215-16-S1-P33

**Published:** 2015-05-29

**Authors:** Aoife M I Waters, Catrin Tudur Smith, Bridget Young, Terry M Jones

**Affiliations:** 1 Department of Biostatistics, Institute of Translational Medicine, University of Liverpool, Liverpool, Merseyside, L69 3GA, UK; 2 Department of Psychological Sciences, Institute of Psychology Health and Society, University of Liverpool, Liverpool, Merseyside, L69 3GB, UK; 3 Department of Molecular and Clinical Cancer Medicine, Institute of Translational Medicine, University of Liverpool, Liverpool, Merseyside, L69 3GA, UK

## Introduction

Contemporary treatments for Oropharyngeal cancer (OPSCC) are associated with a number of debilitating side-effects. Whilst these have a significant impact on the patient’s quality of life, they are often not measured in clinical trials. Additionally there is no standardisation of outcome selection and reporting, even amongst trials of comparable interventions. This reduces the volume of data available for meta-analyses leading to difficulties in both interpreting treatment effect and in making evidence based healthcare decisions. The development of a Core Outcome Set (COS) is proposed to address these problems.

## Methods

1. Systematic review to establish which outcomes are measured in OPSCC trials

2. Qualitative interviews with patients and carers to identify which outcomes they prioritise

3. Delphi study to refine long list of outcomes from review and interviews +/- consensus meeting of key stakeholders to ratify which outcomes should be included in the final COS

## Results

Systematic Review Electronic searches of Pubmed, EMBASE and CENTRAL identified 42 eligible studies. There are significant disparities in the number and type of outcomes measured between trials with a significant focus on outcomes related to survival and disease control. Interviews We recruited 34 patients and carers across 3 centres; two in the UK (NHS Trusts in Liverpool and Sunderland) and one in the US (MD Anderson Cancer Center, Houston, TX). Patients and carers agree that survival outcomes are a priority and must be measured in clinical trials, however functional and health-related quality of life outcomes are also important and should be measured to help patients and clinicians differentiate between treatments. The short and long-term functional and psychological impact of such radical treatments must be considered when making treatment decisions. We will present the findings of the systematic review and interviews and discuss the Delphi Consensus Study development process and results.

